# Electroacupuncture Relieves Fibromyalgia Pain in a Female Mouse Model by Augmenting Cannabinoid Receptor 1 Expression and Suppressing Astrocyte and Microglial Activation in Nociceptive Pathways

**DOI:** 10.3390/biomedicines13092112

**Published:** 2025-08-29

**Authors:** I-Han Hsiao, Ming-Chia Lin, Hsin-Cheng Hsu, Younbyoung Chae, I-Ying Lin, Yi-Wen Lin

**Affiliations:** 1 College of Medicine, School of Medicine, China Medical University, Taichung 404328, Taiwan; 018309@tool.caaumed.org.tw; 2 Department of Neurosurgery, China Medical University Hospital, Taichung 404332, Taiwan; 3 Department of Nuclear Medicine, E-DA Hospital, I-Shou University, Kaohsiung 82445, Taiwan; ed101186@edah.org.tw; 4 Department of Traditional Chinese Medicine, China Medical University Hsinchu Hospital, Hsinchu 302056, Taiwan; 002762@tool.caaumed.org.tw; 5 Acupuncture and Meridian Science Research Center, Kyung Hee University, Seoul 02453, Republic of Korea; ybchae@hu.ac.kr; 6 Department of Anesthesiology, E-DA Hospital, I-Shou University, Kaohsiung 84001, Taiwan; 7 College of Chinese Medicine, Graduate Institute of Acupuncture Science, China Medical University, Taichung 404328, Taiwan; 8 Chinese Medicine Research Center, China Medical University, Taichung 404328, Taiwan

**Keywords:** electroacupuncture, fibromyalgia, CB1, TLR4, microglia, astrocyte

## Abstract

**Background/Objectives**: Fibromyalgia is a chronic pain syndrome with unclear etiology, meaning that it is difficult to treat effectively. The stimulation of cannabinoid receptor 1 (CB1) suppresses neuronal excitability and synaptic transmission in nociceptive pathways via reducing activity in the calcium channel and promoting the opening of the potassium channel. **Methods**: In this study, we examined whether CB1 activity contributes to the antinociceptive efficacy of electroacupuncture (EA) in a mouse fibromyalgia (FM) pain model established using intermittent cold stress (ICS). The model mice demonstrated both mechanical and thermal hyperalgesia measured using the von Frey and Hargreaves tests, respectively. **Results**: Electroacupuncture effectively reduced both forms of hyperalgesia and enhanced CB1 expression in the dorsal root ganglia, spinal cord, hypothalamus, and periaqueductal gray. In addition, EA attenuated the fibromyalgia-associated reactive transformation of microglia and astrocytes and the activation of the pain-related TLR4–MyD88–TRAF6 signaling pathway. The effects of ICS were also mitigated by the deletion of Trpv1, the gene encoding the transient receptor potential cation channel TRPV1 (capsaicin channel) implicated in nociceptive and inflammatory signaling. Further, the antinociceptive efficacy of EA was partially recapitulated by the acupoint injection of a CB1 agonist and abolished by the injection of a CB1 antagonist, suggesting that activating CB1 is essential for this therapeutic effect. **Conclusions**: Electroacupuncture can effectively alleviate mechanical and thermal hyperalgesia in a mouse model affected by fibromyalgia pain by activating the CB1 pathway, highlighting the therapeutic potential of CB1 agonism as a therapeutic strategy.

## 1. Introduction

Fibromyalgia (FM) pain afflicts approximately 2–8% of the global population, and its prevalence is predicted to increase in many regions due to the aging of the population. Diagnosing and treating fibromyalgia are challenging as it is difficult to ascertain the cause of pain, and there is a lack of reliable biomarkers. Pain typically affects the muscles, ligaments, tendons, and soft tissues, particularly in the shoulders, back, chest, arms, and legs [1,2]. Several drugs have been approved by the United States Food and Drug Administration (FDA) for the relief of symptoms, such as duloxetine, pregabalin, and milnacipran, but there are currently no curative treatments, and many cases are difficult to treat with the agents available. Further, for many patients, their pain is not relieved enough, and they can experience adverse side effects [3,4]. Fibromyalgia is highly comorbid with conditions such as depression, anxiety, rheumatoid arthritis, systemic lupus erythematosus, osteoarthritis, and other chronic pain disorders. For these patients, a healthy lifestyle, including the practice of Tai chi, meditation, exercise, and acupuncture, can help to relax the muscles, lower stress levels, and reduce pain symptoms [5,6]. Cordón et al. reported a reduction in the number of inner retinal neurons among fibromyalgia patients, suggesting that neurodegeneration also contributes to disease onset or progression. In addition, patients with fibromyalgia have demonstrated hyperexcitation of the brain and spinal cord neurons, termed central sensitization, concomitant with greater pain intensity or hypersensitivity to normally subthreshold stimuli (hyperalgesia) [7].

Fibromyalgia may also involve the dysregulation of the endogenous cannabinoid system, suggesting that cannabinoids could potentially be used as another treatment. These compounds were first isolated from cannabis leaves, and analogs were subsequently isolated in various tissues (termed endocannabinoids) [8]. CB1 is highly expressed in the brain, especially in the spinal cord, hippocampus, hypothalamus, cerebellum, and periaqueductal gray (PAG), regions that are strongly involved in pain transmission or behavioral responses to pain [9,10]. Endocannabinoid ligands such as 2-arachidonoylglycerol (2-AG) and arachidonylethanolamide (AEA) can bind to CB1 receptors and inhibit adenylate cyclase, leading to reduced intracellular cAMP and PKA activity [11,12]. Additionally, CB1 receptors modulate mitogen-activated protein kinases (MAPKs) such as extracellular signal-regulated kinase (ERK), c-jun N-terminal kinase (JNK), and p38. Alternatively, CB2 receptors primarily regulate immune signaling and may influence neuroinflammation via microglial cells [11,12].

The signaling pathways between neurons and glial cells are crucial drivers of chronic pain progression. Neuronal damage or pathology can induce the phenotypic transformation of microglia and astrocytes to a proinflammatory state, and this reactive transformation is strongly associated with allodynia or hyperalgesia. Microglia in the inflammatory M1 state release proinflammatory cytokines such as tumor necrosis factor α (TNF-α), interferon-γ (IFN-γ), interleukins (ILs), and chemokines that ultimately initiate hyperalgesia and chronic pain [13,14]. High mobility group protein B1 (HMGB1) and S100 calcium-binding protein B (S100B) are two additional signaling factors released from microglia and astrocytes that may contribute to the induction of chronic pain. Both are ligands for toll-like receptor 4 (TLR4), which, upon activation, enhances the synthesis and release of proinflammatory IL-1, IL-2, IL-6, TNF-α, and IFN-γ via a signaling pathway involving the adaptor proteins MyD88 and TRAF6 [15,16,17].

In Asia, acupuncture has been used for over 3000 years to treat various diseases, including chronic pain. Given its efficacy and safety, acupuncture is now practiced worldwide to treat back, low back, and dental pain, headaches, tennis elbow, and fibromyalgia, among other chronic pain conditions [18,19]. Modern acupuncture involves inserting a fine steel needle into a specific acupoint and delivering a low-frequency mild current. A recent double-blind, randomized, controlled trial demonstrated that EA could relieve chronic pain and major depressive disorder [20]. In addition, EA has antinociceptive effects against inflammatory and neuropathic pain, as well as fibromyalgia, potentially by increasing the release of endogenous opioids, cannabinoids, and adenosine [21,22,23,24,25,26]. Further, EA has been reported to reduce mechanical and thermal hyperalgesia by suppressing TRPV1 signaling and attenuating the release of proinflammatory IL-1β, IL-6, TNFα, and IFN-γ [27].

In the current study, we examined the effects of glial inflammatory transformation, CB1 activity, and TLR4 signaling on fibromyalgia-like pain and the efficacy of EA treatment in an intermittent cold stress (ICS) mouse model. We found that ICS induced both mechanical and thermal hyperalgesia, activated microglia and astrocytes in the dorsal root ganglion (DRG), spinal cord, and PAG, and enhanced the expression levels of HMGB1, S100B, TLR4, MyD88, and TRAF6 in these regions, while EA mitigated all of these responses. Further, the behavioral and molecular signs of fibromyalgia were also depleted by *Trpv1* gene deletion and the injection of a CB1 agonist, while the injection of a CB1 antagonist blocked the therapeutic effects of EA. These findings identify TRPV1 and CB1 signaling pathway components as potential therapeutic targets for fibromyalgia.

## 2. Materials and Methods

### 2.1. Mice and Fibromyalgia Pain Model

The experimental subjects were 8–12-week-old female C57B/L6 wild-type mice (18–20 g) purchased from BioLasc Taiwan Ltd. (Yilan, Taiwan). We used female mice to reflect the higher prevalence of fibromyalgia pain in human females than males. After the mice arrived, they were placed in a home cage with a 12 h light/dark cycle (light 6 a.m. to 6 p.m). The temperature was maintained at 25 °C with 60% moisture. The experiments were approved by the Institute of Animal Care and Use Committee of China Medical University, Taiwan (permit no. CMUIACUC-2024-076), according to the *Guide for the Care and Use of Laboratory Animals* (National Academy Press, Washington, DC, USA). We used the G*Power 3.1.9.7 statistic software program to determine the sample size. Nine mice per group was considered to be the minimum number required for a significant alpha level of 0.05 and a power of 80% to be reached. The investigators were blinded to the group division and data examination. The mice were randomly divided into four groups: normal (Normal); cold-stress-induced fibromyalgia pain (FM); cold-stress-induced fibromyalgia pain with EA (FM + EA); and cold-stress-induced fibromyalgia pain in *Trpv1*^−/−^ mice (FM + *Trpv1*^−/−^). To develop the mouse FM model, the mice were kept in a 4 °C environment, while the Normal mice remained at 25 °C. At 10 a.m. the next day, FM mice were transferred to 25 °C for 30 min before being taken back at 4 °C for 30 min. This procedure was performed until 4 p.m., when they were moved again from 4 p.m. to be exposed to different conditions overnight during the first 3 days.

### 2.2. Nociceptive Behavior Examinations

Mice were placed in separate Plexiglas boxes that were perforated overhead, placed on an elevated horizontal wire mesh stand, covered with a dark cloth, maintained in a silent environment at room temperature (25 °C), and allowed to habituate for 30 min before starting the behavioral test. The experiments were only conducted when the mice were calm and all their feet were placed on the surface, without grooming or sleeping taking place. We used the von Frey filament measuring instrument (IITC Life Science Inc., Irwindale, CA, USA) to increase the pain pressure in the center of the right plantar hind paw of the mice. Maximum pressure was reached when the right hind paw was lifted using the plastic tip and the mouse reflexively withdrew the hind paw. We allowed for 3 min breaks between stimuli. The results were recorded as mechanical sensitivity. The Hargreaves test was used to measure the thermal sensitivity of the mice, and the preparations used were similar to those used for the von Frey test. The subjects were placed in an animal enclosure that separated the mice to limit interaction and covered using a dark cloth. After giving the mice 30 min to get used to this habitat, the experiments were initiated. The IITC Plantar Analgesia Meter (IITC Life Sciences, SERIES8, Model 390G) (Irwindale, CA, USA) was used to measure the withdrawal latency time of the mice subjected to radiant heat applied on the surface, targeting the center of the right hind paw.

### 2.3. Electroacupuncture

After being anesthetized with 5% isoflurane for the induction, the mice were maintained via inhaling 1% isoflurane via a head-stuffed tube. A pair of 1” acupuncture needles (32G, Yu Kuang Chem. Ind. Corp., Tainan City, Taiwan) were implanted bilaterally into the ST36 acupoint. The ST36 acupoint in mice is 3–4 mm below the patella, between the fibula and tibia, and on the anterior side of the anterior tibial muscle. The acupoint was electrically stimulated with a constant square pulse with 1 mA intensity, 2 Hz frequency, and 100 μs width for 20 min from a Trio 300 stimulator (Ito, Tokyo, Japan). The EA treatment sessions were performed on days 3 and 4.

### 2.4. Western Blot Analysis

Proteins were extracted from the DRG, SC, thalamus, SSC, and CB of the mice. The protein extracts were prepared; then, 10% radioimmunoprecipitation (RIPA) lysis buffer (Fivephoton Biochemicals, San Diego, CA, USA, RIPA-50), 100 μL of a protease inhibitor (Bionovas, Beijing, China, FC0070-0001), and 100 μL of phosphatase inhibitor (Bionovas, FC0050-0001) were added to the lysate. After homogenization, the mixture was centrifuged at 10,000 rpm for 10 min at 4 °C in an adjustable centrifuge. A total of 10 μL of extracted tissue sample was subjected to 8% or 12% SDS Tris-glycine gel electrophoresis, according to the protein size. A current electrophoresis power supply (PowerPac, Bio-Rad Laboratories, Inc., Singapore) was used to run the gels in two different sections: Section 1 for 40 min at 50 V and Section 2 for 1 h and 50 min at 100 V. A semidry transfer machine (Trans-Blot SD Cell, Bio-Rad Laboratories, Inc., Hercules, CA, USA) transferred the gels onto PVDF membranes at 15 V for 45 min. The transferred membranes were washed with phosphate-buffered saline Tween (PBST with 0.05% Tween20) and blocked with bovine serum albumin for 30 min at 4 °C. Thereafter, the membranes were cultured with primary antibodies in PBST with 1% BSA and incubated overnight at 4 °C. The GFAP (~50 kDa, 1:1000, cat no. AB5804, Merck KGaA, Darmstadt, Germany), Iba1 (~17 kDa, 1:1000, cat no. ACS-010, Alomone, Jerusalem, Israel), HMGB1 (~28 kDa, 1:1000; cat no. SAB210867, Merck), S100B (~10 kDa, 1: 1000; cat no. S2532, Merck), CB1 (~45kDa, 1:1000, Alomone, Jerusalem, Israel), TLR4 (~35 kDa, 1:1000, cat no. MABF2274, Merck), MyD88 (~35 kDa, 1:1000, cat no. AB16527, Merck), and TRAF6 (~87 kDa, 1:1000, cat no. 23-059, Merck) markers were assessed. Then, the membranes were incubated with secondary antibodies at 1:5000 in peroxidase-conjugated goat anti-rabbit antibody (Jackson Immuno Research Laboratory, goat anti-mouse antibody (Jackson Immuno Research Laboratory, West Grove, PA, USA)) for 2 h at 25 °C. Finally, we used an enhanced chemiluminescence substrate kit (PIERCE, Thermo Fisher Scientific Inc., Waltham, MA, USA) to visualize the protein bands on the membranes with LAS-3000 Fujifilm (Fuji Photo Film Co., Ltd., Tokyo, Japan). The image density levels of the specific protein bands were quantified using NIH ImageJ 1.54 h software (Bethesda, MD, USA). α-Tubulin was used as the internal controller.

### 2.5. CB1 Receptor Agonist and Antagonist Administration

Adult C57BL/6 female mice (*n* = 6) were used for the CB1 agonist or antagonist test. After fibromyalgia pain was introduced to the mice, 1 μL of CB1 agonist AEA (Sigma, St. Louis, MO, USA) was administered at the acupoint or i.c.v at 100 μM. Alternatively, 1 μL of CB1 antagonist AM251 (Sigma, St. Louis, MO, USA; in 10 μL of saline) was immediately administered at the acupoint or i.c.v at 5 μg. Under light isoflurane anesthesia (1%), AEA and AM251 were administered after the induction of fibromyalgia pain.

### 2.6. Statistical Analysis

Statistical analysis was performed using SPSS 21.0, a statistical program. Statistical data are presented as the mean ± standard error of the mean (SEM). Differences among the groups were analyzed using a one-way ANOVA test, followed by Tukey’s post hoc test. *p* < 0.05 was considered statistically significant.

## 3. Results

### 3.1. Electroacupuncture (2 Hz) and Trpv1 Gene Knockout Diminished Mechanical and Thermal Hyperalgesia in Intermittent Cold Stress (ICS)-Induced Fibromyalgia Model Mice

**Figure 1:**
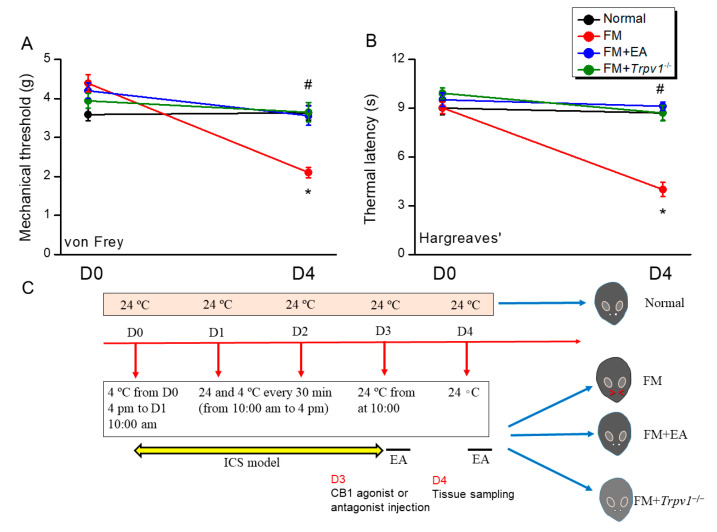
Electroacupuncture or *Trpv1* knockout suppressed fibromyalgia-like mechanical and thermal hyperalgesia induced via intermittent cold stimulation (ICS) in mice. (**A**) Mechanical hyperalgesia, as measured using the von Frey filament test. (**B**) Thermal hyperalgesia, as measured using the Hargraves test. (**C**) Study design and ICS protocol. Normal: normal mice; FM: fibromyalgia model mice; FM + EA: fibromyalgia model mice treated with EA; FM + *Trpv1*^−/−^: fibromyalgia model mice on the *Trpv1*^−/−^ background. * *p* < 0.05 vs. Normal group. ^#^ *p* < 0.05 vs. FM group. *n* = 9.

To examine the efficacy of EA against fibromyalgia-like mechanical and thermal hyperalgesia and the potential effects of CB1 channel signaling, we established ICS-induced fibromyalgia model mice on the wild-type and *Trpv1*^−/−^ backgrounds and conducted von Frey hair and Hargreaves tests (Figure 1). Fibromyalgia model mice (FM group) demonstrated a lower mechanical withdrawal threshold compared with the untreated Normal group mice (Figure 1A, red circle vs. black circle, day 4: 2.1 ± 0.13 g vs. 3.63 ± 0.18, *n* = 9 mice per group), indicating the successful induction of mechanical hyperalgesia. The administration of 2 Hz EA (FM + EA group) significantly increased the withdrawal threshold, which was consistent with the reduced mechanical hyperalgesia, as previously described (Figure 1A, blue circle, day 4: 3.56 ± 0.25 g, *n* = 9 mice per group). Furthermore, no mechanical hyperalgesia was observed in ICS model mice on the *Trpv1*^−/−^ background (Figure 1A, green circle, day 4: 3.65 ± 0.24 g, *n* = 9 mice per group). The model mice also demonstrated significant thermal hyperalgesia in the Hargreaves test (Figure 1B, red circle, day 4: 3.99 ± 0.44 s, *n* = 9 mice per group), which was again reduced via 2 Hz EA (Figure 1B, blue circle, day 4: 9.13 ± 0.25 s, *n* = 9 mice per group) and absent in the ICS mice established on the *Trpv1*^−/−^ background (Figure 1B, green circle, day 4: 8.71 ± 0.47 s, *n* = 9 mice per group). These findings indicate that the stimulation of *Trpv1* is essential for ICS-induced hyperalgesia induction and that this response can be mitigated via EA. The study design and ICS protocol are shown in Figure 1C.

### 3.2. Both EA and Trpv1 Knockout Reduced Fibromyalgia-like Pain in ICS Model Mice by Suppressing Microglial and Astrocytic Activation and Promoting CB1 Pathway Activity

**Figure 2:**
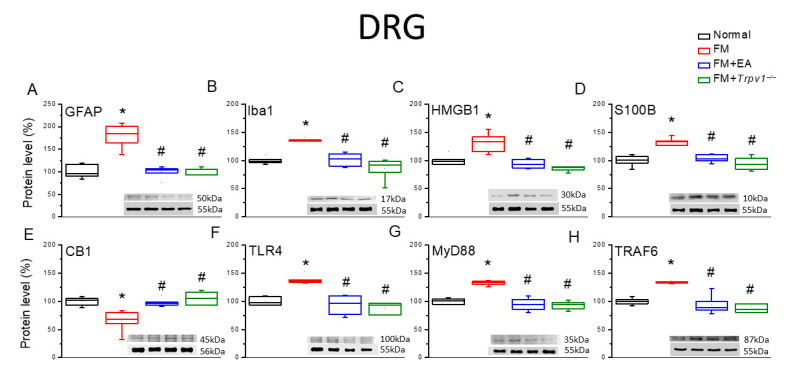
Expression levels of CB1, astrocyte and microglia markers, and nociceptive signaling molecules in the DRG of the four mouse treatment groups (Normal, FM, FM + EA, and FM + *Trpv1*^−/−^), as measured using Western blotting. There are significant increases in (**A**) GFAP, (**B**) Iba1, (**C**) HMGB1, (**D**) S100B, (**E**) CB1 (decrease), (**F**) TLR4, (**G**) MyD88, and (**H**) TRAF6. These increases can be attenuated via EA or *Trpv1* gene deletion. * *p* < 0.05 vs. the Normal group; ^#^ *p* < 0.05 vs. the FM group. *n* = 6.

The induction of chronic pain states is frequently accompanied by the reactive transformation of microglia and astrocytes into proinflammatory phenotypes. To assess the effects of reactive transformation on the model’s induction and therapeutic responses, we estimated the expression levels of marker proteins for reactive transformation 4 days after fibromyalgia induction (Figure 2). The expression levels of both the astrocyte marker GFAP and the microglial marker Iba1 were significantly enhanced in the DRG tissue of FM group mice compared with Normal group mice (Figure 2A,B, * *p* < 0.05, *n* = 6), and these responses were attenuated by 2 Hz EA and *Trpv1* gene deletion (FM + EA and FM + *Trpv1*^−/−^ groups). Further, the expression levels of HMGB1 and S100B were also upregulated in the DRG of FM group mice (Figure 2C,D, * *p* < 0.05, *n* = 6), and these responses were reduced or diminished via EA and *Trpv1* knockout (Figure 2C,D, ^#^ *p* < 0.05, *n* = 6). Conversely, the expression level of CB1 was reduced in the DRG of FM group mice compared with the Normal group (Figure 2E, * *p* < 0.05, *n* = 6), while 2 Hz EA and *Trpv1* knockout decreased this reduction (Figure 2E, ^#^ *p* < 0.05, *n* = 6). Consistent with these results, the expression of TLR4 was elevated in the DRG of FM group mice (Figure 2F, * *p* < 0.05, *n* = 6) and suppressed via EA (Figure 2F, ^#^ *p* < 0.05, *n* = 6). Similarly, the expression of the downstream effector molecules MyD88 and TRAF6 was upregulated in the DRG of FM group mice compared with Normal group mice, and the upregulation of both proteins was diminished by 2-Hz EA and *Trpv1* knockout (Figure 2G,H, *n* = 6).

### 3.3. EA and Trpv1 Knockout Suppressed Fibromyalgia-like Hyperalgesia Through the Modulation of Glial Signaling at the Spinal Cord and Hypothalamic and PAG Levels

**Figure 3:**
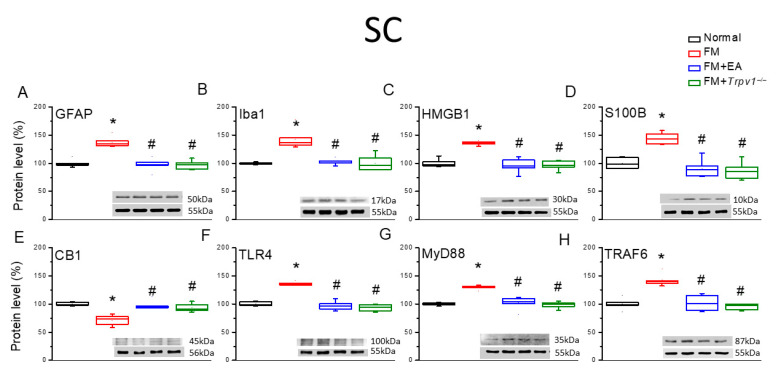
Expression levels of CB1, astrocyte and microglia markers, and nociceptive signaling molecules in the lumbar spinal cord (SC) of the four mouse treatment groups, as measured using Western blotting. There are noteworthy increases in (**A**) GFAP, (**B**) Iba1, (**C**) HMGB1, (**D**) S100B, (**E**) CB1 (decrease), (**F**) TLR4, (**G**) MyD88, and (**H**) TRAF6. These increases can be reduced via EA or *Trpv1* gene deletion. * *p* < 0.05 vs. the Normal group; ^#^ *p* < 0.05 vs. the FM group. *n* = 6.

**Figure 4:**
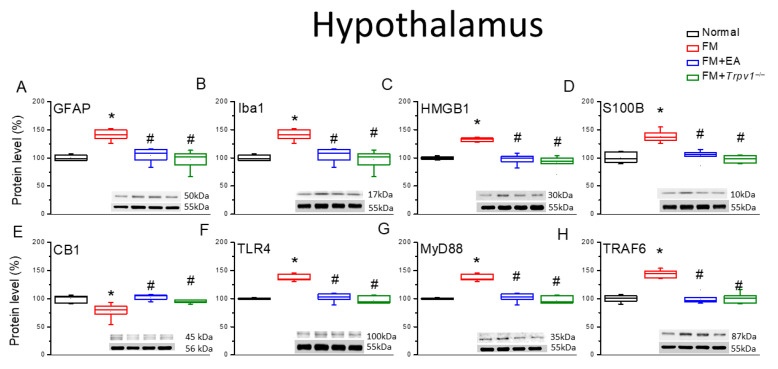
Expression levels of CB1, astrocyte and microglia markers, and nociceptive signaling molecules in the hypothalamus of the four mouse treatment groups. There are significant increases in (**A**) GFAP, (**B**) Iba1, (**C**) HMGB1, (**D**) S100B, (**E**) CB1 (decrease), (**F**) TLR4, (**G**) MyD88, and (**H**) TRAF6. The augmentation can be mitigated via EA or *Trpv1* gene deletion. * *p* < 0.05 vs. the Normal group; ^#^ *p* < 0.05 vs. the FM group. *n* = 6.

**Figure 5:**
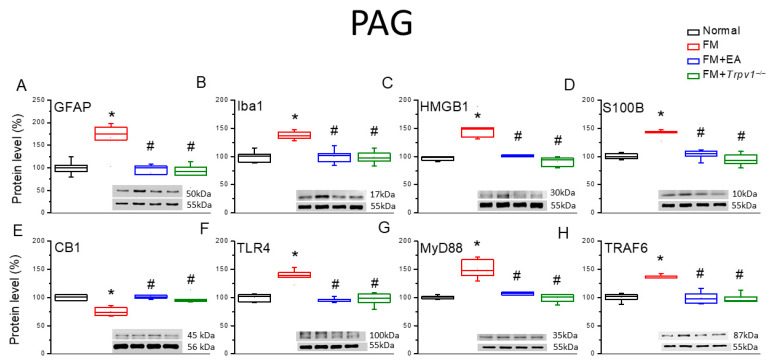
Expression levels of CB1, astrocyte and microglia markers, and nociceptive signaling molecules in the periaqueductal gray (PAG) of the four mouse treatment groups. There are significant increases in (**A**) GFAP, (**B**) Iba1, (**C**) HMGB1, (**D**) S100B, (**E**) CB1 (decrease), (**F**) TLR4, (**G**) MyD88, and (**H**) TRAF6. These rises can be diminished via EA or *Trpv1* gene deletion. * *p* < 0.05 vs. the Normal group; ^#^ *p* < 0.05 vs. the FM group. *n* = 6.

Similarly to DRG, the expression levels of the astrocyte and microglial markers GFAP and Iba1 were elevated in the lumbar spinal cord (SC) tissue of FM group mice compared with the Normal group (Figure 3A,B, * *p* < 0.05, *n* = 6), and the upregulation of both markers was attenuated by 2 Hz EA and in *Trpv1*^−/−^ model mice (FM + *Trpv1*^−/−^) (Figure 3). In addition, the expression levels of HMGB1, S100B, TLR4, MyD88, and TRAF6 were upregulated in the lumbar SC of FM group mice compared with the Normal controls (Figure 3, * *p* < 0.05, *n* = 6), and upregulation was again decreased by the 2 Hz EA treatment and *Trpv1* knockout (Figure 3, ^#^ *p* < 0.05, *n* = 6). Conversely, the expression of CB1 was downregulated by ICS (FM group vs. Normal group) and upregulated by 2 Hz EA and *Trpv1* knockout (Figure 3E, ^#^ *p* < 0.05, *n* = 6). Qualitatively identical results were obtained from hypothalamic and PAG tissues (Figure 4 and Figure 5).

### 3.4. EA Significantly Reduced Fibromyalgia-like Hyperalgesia Through CB1 Receptor Activation

**Figure 6:**
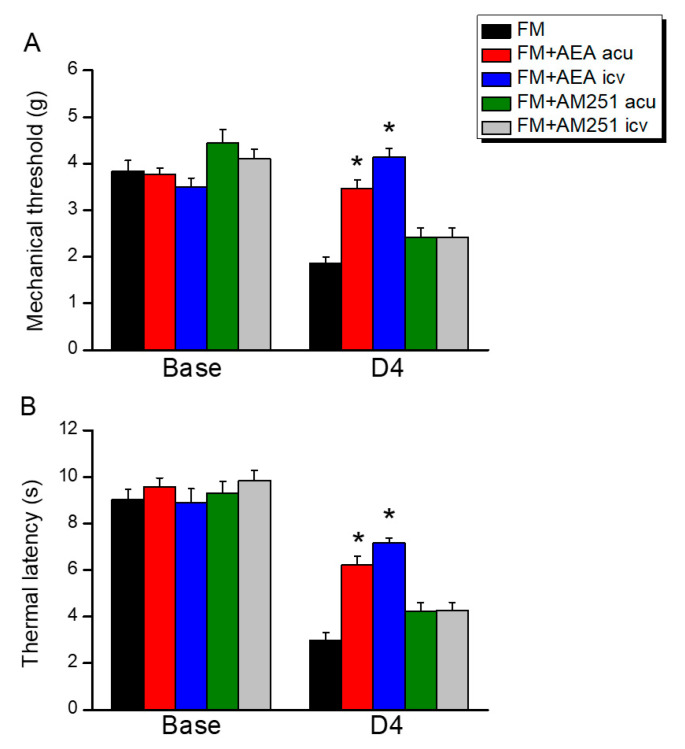
Effects of the CB1 agonist AEA and antagonist AM251 on fibromyalgia-like hyperalgesia in mice. (**A**) Mechanical hyperalgesia and (**B**) thermal hyperalgesia were attenuated via AEA injection. EA analgesia was then diminished via AM251 injection. acu = acupoint injection. icv = intracerebral ventricle injection. * *p* < 0.05 vs. FM group at D4. *n* = 9.

The von Frey test demonstrated that ICS induced fibromyalgia-like mechanical hyperalgesia and that this effect was associated with the downregulation of CB1 receptors in the DRG, SC, hypothalamus, and PAG, while the mitigation of hyperalgesia via EA and *Trpv1* knockout was associated with the reversal of CB1 receptor downregulation. Consistent with these results, the injection of the CB1 agonist AEA at either the peripheral acupoint or by ICV eliminated mechanical hyperalgesia in the absence of EA or *Trpv1* knockout (Figure 6). Conversely, the administration of the CB1 receptor antagonist AM251 at the acupoint or via ICV reversed the effect of EA on hyperalgesia. Qualitatively identical results were obtained for thermal hyperalgesia.

### 3.5. Distinct Downstream Mechanisms for EA-Induced CB1-Mediated Suppression of Fibromyalgia-like Hyperalgesia in the Peripheral and Central Nervous Systems

**Figure 7:**
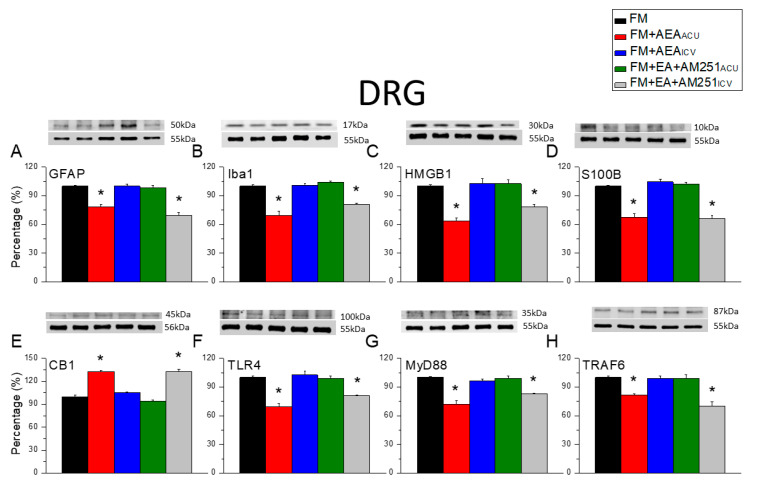
Changes in CB1, glial marker, and nociceptive signaling factor expression levels in mouse DRG induced by acupoint and ICV injection of AEA or AM251. There are significant decreases in (**A**) GFAP, (**B**) Iba1, (**C**) HMGB1, (**D**) S100B (**E**) CB1 (increase), (**F**) TLR4, (**G**) MyD88, and (**H**) TRAF6 in FM + AEA_acu_ and FM + EA + AM251_icv_ groups. Black: FM group; red: FM treated with AEA at the acupoint; blue: FM group treated with AEA via ICV injection; green: FM group treated with EA and AM251 at the acupoint; grey: FM treated with EA and AM251 via ICV injection. * *p* < 0.05 vs. the FM group. *n* = 6.

**Figure 8:**
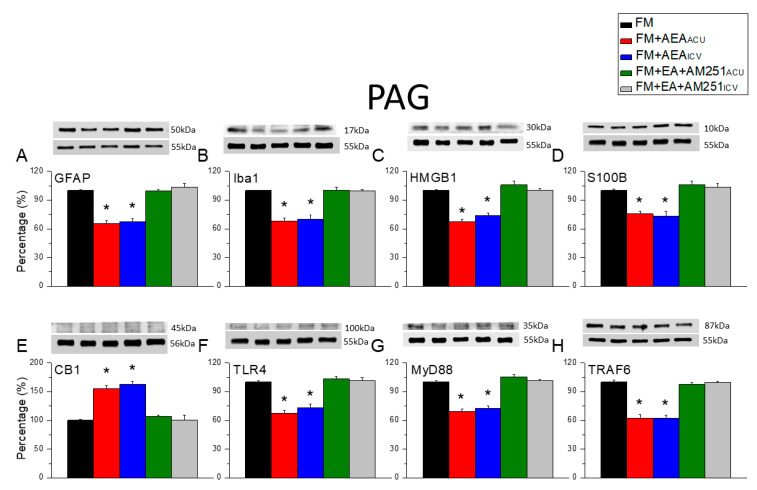
Changes in CB1, glial marker, and nociceptive signaling factor expression levels in mouse PAG induced by acupoint and the ICV injection of AEA or AM251. There are significant decreases in (**A**) GFAP, (**B**) Iba1, (**C**) HMGB1, (**D**) S100B (**E**) CB1 (increase), (**F**) TLR4, (**G**) MyD88, and (**H**) TRAF6 in the FM + AEA_acu_ and FM + AEA_icv_ groups. Black: FM group; red: FM group treated with AEA at the acupoint; blue: FM group treated with AEA via ICV injection; green: FM group treated with EA and AM251 at acupoint; grey: FM group treated with EA and AM251 via ICV injection. * *p* < 0.05 vs. the FM group. *n* = 6.

We next examined whether similar downstream signaling pathways are involved in the peripheral and central effects of EA (Figure 7 and Figure 8). Western blotting revealed that the reversal of astrocyte and microglial marker upregulation in the DRG via EA was recapitulated via acupoint injection of the CB1 agonist AEA (Figure 7A,B, * *p* < 0.05, *n* = 6). In other words, the acupoint injection of AEA reduced the expression levels of glial activation markers compared with the baseline in the DRG of FM group mice. Alternatively, the ICV injection of AEA did not alter marker expression in the DRG compared with the baseline. The reduction in marker expression induced via EA was blocked by the acupoint injection of the antagonist AM251 but not via ICV injection (Figure 7A,B, * *p* < 0.05, *n* = 6). Similarly, S100B and HMGB1 upregulation in the DRG of FM model mice was diminished by the acupoint injection of AEA (Figure 7C,D, * *p* < 0.05, *n* = 6) but not via ICV injection. Reductions in the expression of S100B and HMGB1 induced via EA were prevented by the acupoint injection of AM251 (Figure 7C,D, * *p* < 0.05, *n* = 6) but not via ICV injection. The acupoint injection of AEA significantly augmented CB1 expression in the DRG of FM model mice (i.e., recapitulated the effect of EA), while ICV injection had no effect on the DRG expression of CB1. The acupoint injection of AM251 in FM mice receiving EA blocked CB1 upregulation, while the ICV injection again had no effect (i.e., CB1 was still upregulated). Finally, the TLR4, MyD88, and TRAF6 expression levels in the DRG of FM model mice were downregulated by the acupoint injection of AEA (recapitulating the effect of EA) but not via ICV injection, and, again, the acupoint injection of AM251 (Figure 7F–H, * *p* < 0.05, *n* = 6) but not the ICV injection of AM251 reversed the reductions in the DRG expression of these pain signaling proteins via EA.

We then examined the central effects of EA and the effects of the CB1 stimulation by measuring the expression levels of the same proteins in the PAG (Figure 8). Both the GFAP and Iba1 expression levels were elevated in the PAG of fibromyalgia model mice (Figure 8A,B, * *p* < 0.05, *n* = 6), but in contrast to the DRG, these responses were depleted by both acupoint and the ICV injection of AEA (i.e., both acupoint and ICV injection recapitulated the effects of EA). In addition, both acupoint and the ICV injection of AM251 diminished marker downregulation via EA in FM model mice, indicating that these effects were mediated by central CB1 receptors. The expression levels of S100B and HMGB1 were also higher in the PAG of FM model mice (Figure 8C,D, * *p* < 0.05, *n* = 6), and this response was attenuated by both acupoint and the ICV injection of the CB1 agonist AEA. Again, the reductions in S100B and HMGB1 expression via EA were mitigated by both acupoint and the ICV injection of the CB1 antagonist AM251. Similarly, both acupoint and the ICV injection of AEA enhanced CB1 expression in FM model mice (Figure 8E, * *p* < 0.05, *n* = 6), while CB1 upregulation following EA was reversed by both acupoint and the ICV injection of AM2521. Finally, both acupoint and the ICV injection of AEA suppressed the expression of TLR4, MyD88, and TRAF6 in the PAG (Figure 8F, * *p* < 0.05, *n* = 6), and the suppressive effects of EA on the expression levels were depleted by acupoint and the ICV injection of AM251. All raw data were presented as Table S1.

## 4. Discussion

Abd-ellatief et al. reported that peripheral hyperalgesia following the intramuscular injection of acid saline was accompanied by the proinflammatory signaling factors IL-1β, IL-6, and TNF-α having increased serum concentrations. In the nervous system, astrocytes and microglia are central mediators of inflammatory responses, suggesting that immune activation of these cells contributes to hyperalgesia in chronic pain conditions. Indeed, light and electron microscopy studies of FM model rats have revealed neurodegeneration and astrogliosis in the hippocampus accompanied by the augmented expression of the reactive transformation markers GFAP and inducible nitric oxide synthase (iNOS). These responses were depleted by the α2-adrenergic receptor agonist and analgesic compound dexmedetomidine [28]. Astrocytes also modulate glutamatergic transmission and protect neurons from degeneration through the regulation of extracellular ions and perisynaptic glutamate concentrations. In response to nerve injury, astrocytes may proliferate, enlarge, and release cytokines and chemokines such as HMGB1 and S100B, which can in turn induce the sensitization of nociceptive neurons. Similarly, chemical activation of astrocytes by lipopolysaccharide or TNFα injection can induce mechanical hyperalgesia. Zhang et al. reported that the administration of OR486, an inhibitor of the catecholaminergic metabolic enzyme catechol-O-methyltransferase (COMT), induced mechanical hypersensitivity accompanied by augmented astrocyte activation, and they also found that peripheral β-adrenergic receptors increased the synthesis of proinflammatory cytokines and the activation of astrocytes at the spinal level [29]. Hsiao et al. provided detailed evidence that the analgesic effect of oral eicosapentaenoic acid (EPA) in fibromyalgia model mice was associated with suppressed astrocyte activation and the reduced release of proinflammatory signaling factors, including HMGB1 and S100B. They further confirmed that the augmentation of the expression of toll-like receptor 4 in the mouse cerebellum can be alleviated via the intake of EPA [30].

The activation of microglia is also strongly implicated in fibromyalgia. Albrecht et al. reported widespread microglia activation in the cortex of fibromyalgia patients compared with the Normal subjects, as revealed by positron emission tomography (PET) using [11C]PBR28 as a tracer for the activated microglial marker translocator protein (TSPO). The fatigue observed in patients with fibromyalgia was also associated with hypertrophy of the anterior and posterior middle cingulate cortices, which is, again, consistent with neuroinflammation and microglia activation [31]. Fülöp et al. reported that mechanical hyperalgesia in female mice subjected to chronic restraint stress (CRS) was dramatically attenuated via IL-1 knockout. Further, CRS amplified the number of Iba1-positive cells in the central nucleus of the amygdala, SSC, and PAG of wild-type mice but not IL-1 knockout mice [32]. Wakatsuki et al. reported that repeated cold stress (RCS)-induced chronic pain in mice was associated with the upregulation of the injury marker activating transcription factor (ATF3) in the lumbar DRG. Activated microglia were also found in the medial part and ventral horn of the nucleus proprius [33]. In accordance with the seminal effects of astroglial and microglial activation on fibromyalgia pain, we found that both EA or *Trpv1* gene deletion concomitantly attenuated behavioral pain responses and both astrocyte and microglia activation in the DRG, SC, hypothalamus, and PAG of a fibromyalgia mice model.

A recent study of 2000 fibromyalgia patients found that over 60% used cannabinoids to relieve their pain and alleviate their symptoms. While cannabinoids can relieve pain conditions, about half of patients experience side effects [34]. Yassin et al. reported that medical cannabinoid treatment for >6 months following 3 months of standard therapy considerably improved symptoms in a cohort 31 patients [35]. A double-blind, randomized, placebo-controlled clinical trial using the fibromyalgia impact questionnaire also indicated that cannabinoids provide therapeutic benefits without intolerable side effects [36]. Yen et al. reported that EA can diminish chronic fibromyalgia pain through the attenuation of the transient receptor potential vanilloid 1 signaling pathway in the mouse thalamus and somatosensory cortex. They injected 20 μL of acidic saline (pH 4.0) into the right gastrocnemius muscle and found that there was chronic fibromyalgia pain with increased protein kinase A, pERK, and pCREB [37]. In the current study, EA similarly attenuated fibromyalgia-like pain and concomitantly upregulated CB1 receptor expression and downstream signaling factors in the DRG, SC, hypothalamus, and PAG without significant side effects. Moreover, these therapeutic effects of EA were abolished by pharmacological CB1 blockade, further supporting the role that CB1 receptor signaling played in the analgesic effect of EA.

The administration of LPS to healthy individuals with moderately severe fibromyalgia augmented leptin levels and repressed fractalkine levels, as measured using a multi-cytokine ELISA. In addition, fibromyalgia patients displayed lower concentrations of IFN-γ, IL-12, and IL-17A compared with the healthy controls, consistent with immunoreactions induced via TLR4 signaling [38]. It was also reported that the activation of TLR4 by lysozyme induced TRIF but not MyD88 and further triggered weak inflammatory cytokine expression and augmented glutamate release, suggesting the existence of a MyD88-independent pathway for nociceptive progression in fibromyalgia [39]. Si et al. found that the suppression of LPS-induced mechanical and thermal hyperalgesia in neuropathic pain model rats via oral anti-inflammatory stigmasterol was accompanied by increased serum concentrations of IL-1β, IL-8, and TGF-β; the significant attenuation of Iba1; TLR4, MyD88, and pNF-κB upregulation in the spinal cord; and a shift in the microglial phenotype from M1 to M2 polarization [40]. The CB1 receptor was reported to inhibit TRPV1 and the associated Ca^2+^ influx, contributing to the analgesic effect in both normal and inflamed conditions. Agonists active at the CB1 receptor were found to be beneficial in inflammatory nociception conditions [41]. Similarly, we found that EA treatment or *Trpv1* gene deletion reversed the overexpression of TLR4, MyD88, and TRAF6, and this effect was blocked by CB1 receptor antagonists.

## 5. Conclusions

The current study revealed that the suppression of fibromyalgia-like pain in a mouse model via EA requires the activation of the CB1–TLR4 signaling pathway, the mitigation of glial activation, and the suppression of the TLR4–MyD88–TRAF6 signaling pathway. Indeed, both mechanical and thermal hyperalgesia were attenuated by *Trpv1* knockout in addition to EA, while the analgesic effect of EA was associated with the upregulation of CB1 receptors in the DRG, SC, hypothalamus, and PAG. The administration of a CB1 antagonist attenuated EA analgesia. Collectively, these findings highlight the potential therapeutic value of CB1 pathway modulation for fibromyalgia pain. The limitation of this study is that we used only female mice as experimental subjects for the FM model. In future, studies should include male mice as well as female mice in order for these findings to be generalized to other sexes. We suggest that future research implements these recommendations in order for EA to be established as an FM treatment.

## Data Availability

The original contributions presented in this study are included in the article. Further inquiries can be directed to the corresponding authors.

## Supplementary Materials

The following supporting information can be downloaded at: https://www.mdpi.com/article/10.3390/biomedicines13092112/s1, Table S1. Valuation of protein percentages (%) in DRG and PAG across five groups. The FM group was set as 100% and served as a reference. Statistical differences were surveyed using an analysis of variance test, followed by a post hoc Tukey’s test. *signifies a significant difference, compared to the FM group (*p* < 0.05).
