# Supplementary material for: Electroacupuncture Relieves Fibromyalgia Pain in a Female Mouse Model by Augmenting Cannabinoid Receptor 1 Expression and Suppressing Astrocyte and Microglial Activation in Nociceptive Pathways
Source: Biomedicines. 2025 Aug 29;13(9):2112. doi: 10.3390/biomedicines13092112 (PMC12467102; doi:10.3390/biomedicines13092112)
Supplement: Supplementary file 1 [file biomedicines-13-02112-s001.zip › biomedicines-3749478-supplementary.pdf]

Table S1. Valuation of protein percentages (%) in DRG and PAG across five groups. The FM group was set as 100% and served as a reference. Statistical differences were surveyed using an analysis of variance test, followed by a post hoc Tukey's test. *signifies a significant difference, compared to the FM group ($p < 0.05$).

Region		GFAP		Iba1		HMGB1		S100B		CB1		TLR4		MyD88		TRAF6	
		Mean	SE	Mean	SE	Mean	SE	Mean	SE	Mean	SE	Mean	SE	Mean	SE	Mean	SE
DRG	FM	100.0	1.1	100.0	1.6	100.0	1.0	100.0	3.1	100.0	1.8	100.0	1.8	100.0	0.9	100.0	1.7
	*FM+AEA_{ACU}	78.2	2.5	69.4	4.2	67.7	3.8	63.8	2.7	132.6	1.5	69.4	2.9	71.9	4.0	81.8	1.1
	FM+AEA_{ICV}	100.0	2.1	100.7	1.8	104.6	2.7	102.9	5.4	105.3	0.9	102.6	3.7	96.3	2.3	99.0	2.8
	FM+EA+$AM251_{ACU}$	98.4	2.5	103.9	1.4	102.2	2.0	102.6	4.3	94.2	1.3	99.2	2.2	99.1	2.6	98.7	4.3
	*FM+EA+$AM251_{ICV}$	69.4	3.5	80.6	1.6	66.4	2.7	78.6	2.6	132.6	3.3	81.1	0.6	82.6	0.7	70.1	4.4
PAG	FM	100.0	1.3	100.0	0.5	100.0	1.5	100.0	1.2	100.0	1.8	100.0	1.3	100.0	1.3	100.0	2.1
	*FM+AEA_{ACU}	65.2	3.2	67.9	3.5	75.5	2.7	67.5	2.5	155.3	5.3	67.3	3.3	69.4	2.5	62.4	3.7
	*FM+AEA_{ICV}	67.2	3.5	70.0	4.1	73.1	5.5	74.0	2.9	163.3	4.9	73.2	3.8	72.9	2.2	62.5	2.9
	FM+EA+$AM251_{ACU}$	99.4	1.7	100.1	3.4	106.0	4.0	106.0	3.9	106.6	2.9	103.6	2.6	105.4	2.4	97.7	2.0
	FM+EA+$AM251_{ICV}$	103.2	4.2	99.6	1.4	103.5	3.7	100.1	2.0	101.1	8.3	101.9	2.8	101.4	1.6	99.6	1.6
